# Enzymatic Activity and Its Relationships with the Total Phenolic Content and Color Change in the High Hydrostatic Pressure-Assisted Curing of Vanilla Bean (*Vanilla planifolia*)

**DOI:** 10.3390/molecules28227606

**Published:** 2023-11-15

**Authors:** Génesis V. Buitimea-Cantúa, Viridiana Chávez-Leal, Mayra C. Soto-Caballero, Dario I. Tellez-Medina, Jorge Welti-Chanes, Zamantha Escobedo-Avellaneda

**Affiliations:** 1 Tecnologico de Monterrey, Escuela de Ingeniería y Ciencias, Ave. Eugenio Garza Sada 2501, Monterrey 64849, Mexico; 2 Facultad de Ciencias Agrotecnologicas, Universidad Autónoma de Chihuahua, Av. Presa de la Amistad 2015, Cuauhtémoc, Chihuahua 31510, Mexico; 3 Instituto Politécnico Nacional, Escuela Nacional de Ciencias Biológicas, Prolongación de Carpio y Plan de Ayala S/N, Casco de Santo Tomás, Azcapotzalco, Ciudad de México 11340, Mexico

**Keywords:** high hydrostatic pressure (HHP), peroxidase (POD), polyphenol oxidase (PPO), total phenolics (TPC), color, vanilla curing

## Abstract

Diverse enzymatic reactions taking place after the killing of green vanilla beans are involved in the flavor and color development of the cured beans. The effects of high hydrostatic pressure (HHP) at 50–400 MPa/5 min and blanching as vanilla killing methods were evaluated on the total phenolic content (TPC), polyphenoloxidase (PPO), and peroxidase (POD) activity and the color change at different curing cycles of sweating–drying (C0–C20) of vanilla beans. The rate constants describing the above parameters during the curing cycles were also obtained. The TPC increased from C1 to C6 compared with the untreated green beans after which it started to decrease. The 400 MPa samples showed the highest rate of phenolic increase. Immediately after the killing (C0), the highest increase in PPO activity was observed at 50 MPa (46%), whereas for POD it was at 400 MPa (25%). Both enzymes showed the maximum activity at C1, after which the activity started to decrease. As expected, the L* color parameter decreased during the entire curing for all treatments. An inverse relationship between the rate of TPC decrease and enzymatic activity loss was found, but the relationship with L* was unclear. HHP appears to be an alternative vanilla killing method; nevertheless, more studies are needed to establish its clear advantages over blanching.

## 1. Introduction

Vanilla (*Vanilla planifolia*) is an important crop native to Mexico. During the curing of flavorless green vanilla beans, a complex mixture of more than 200 components is produced, and the beans acquire a dark brown color. Among them, vanillin (4-hydroxy-3-methoxy-benzaldehyde) is the main compound responsible for flavor [1]. The curing of vanilla beans is a long, traditional, and uncontrolled process that involves four stages: killing, sweating, sun drying, and conditioning [2]. Sweating and drying are performed simultaneously in multiple sweating–drying cycles until the beans reach a moisture content of approximately 20–30%. Vanilla killing is traditionally achieved by immersing green beans in hot water for a short time to stop the vegetative phase promoting physical and metabolic changes that release enzymes and substrate necessary for flavor development [1]. Thus, high enzymatic activities are desirable to promote the required biochemical reactions. Yet, killing by blanching does not cause great changes in the microstructure of the beans but decreases enzymatic activity [3,4]. Therefore, the use of alternative killing methods that alter the cellular structure without affecting the enzyme activity of vanilla beans is necessary.

Recently, vanilla killing using high hydrostatic pressure (HHP), a nonthermal technology, was explored by applying 50–600 MPa to promote the hydrolysis of β-*d*-glucovanillin by β-*d*-glucosidase to form vanillin. The results showed that HHP improved the vanillin content and increased the β-*d*-glucosidase activity; thus, it was proposed as a suitable option for the nonthermal killing of vanilla beans [5]. The positive impact on enzymes and phenolic compounds was attributed to the effect of HHP on the cell membrane and the unfolding of proteins. In the first effect, the change in membrane permeability produces the release of enzymes and substrates from cell compartments. In this way, the enzyme–substrate interaction is facilitated, which could result in an increment in the enzyme activity. In the second effect, the conformational change in the protein eventually leads to a change in the enzyme activity (increment or decrement) depending on the treatment conditions [6].

After vanilla killing and during the subsequent curing stages (i.e., sweating, drying, and conditioning), vanillin and other phenolics are released which act as precursors of other metabolites, among them those responsible for the characteristic flavor and dark brown color of cured beans. In vanilla beans, the dark desirable color is mainly developed by enzymatic and nonenzymatic browning reactions. Two main enzymes are involved in enzymatic browning reactions, polyphenoloxidase (PPO) and peroxidase (POD), which are associated with the de-greening of vanilla beans [7]. PPO is a copper-oxidoreductase, which, in the presence of oxygen, catalyzes the hydroxylation of monophenols to diphenols and the oxidation of catechol to quinones, which, subsequently, are mostly polymerized to dark pigments. POD utilizes H_2_O_2_ and other hydroperoxides, as well as O_2_, to catalyze the oxidation of various cellular substrates, including the oxidation of aromatic compounds. It has been reported that PPO and POD act synergistically during enzymatic browning: while in the presence of molecular oxygen, PPO generates hydrogen peroxide from the oxidation of phenols producing quinones; POD uses these compounds as substrates to produce dark brown pigments [7,8].

The application of mathematical modeling through kinetic-type parameters to describe the degradation rates of food during processing and storage is important for the optimization of complex processes and the prediction of shelf life [9,10]. Kinetic analysis is a valuable tool to compare the impact of different process technologies on enzymatic activity (for analysis of activation/inactivation), change in compounds concentration, and color of various fruits and vegetables [11]. Enzymatic activity and nonenzymatic browning, among other reactions, have been adequately fitted to zero-order kinetics [10,12]. Rancidity, vitamin loss, microbial growth, off flavors production, enzymatic activity, and color change have been described using first-order kinetic models [10,11,13,14,15,16,17,18,19,20]. Although less commonly, the second-order model has been used to describe thermal loss of vitamin C [21].

The effect of HHP on β-glucosidase activity [5], as well as the activity of PPO and POD [1], and vanillin concentration at different curing cycles [22] during the traditional curing process of vanilla beans have been investigated; however, to our knowledge, there is no information about PPO, POD activity, TPC, and color change during HHP-assisted curing. This work aimed to evaluate the effect of HHP, as a vanilla killing method, on the activity of PPO and POD, as well as on TPC and color evolution, during different curing cycles (sweating–drying) compared with blanched-treated beans. The rate constants that characterize the enzymatic activity and phenolic formation/degradation under HHP and blanching were also obtained.

## 2. Results and Discussion

### 2.1. Total Phenolic Content (TPC)

**Figure 1:**
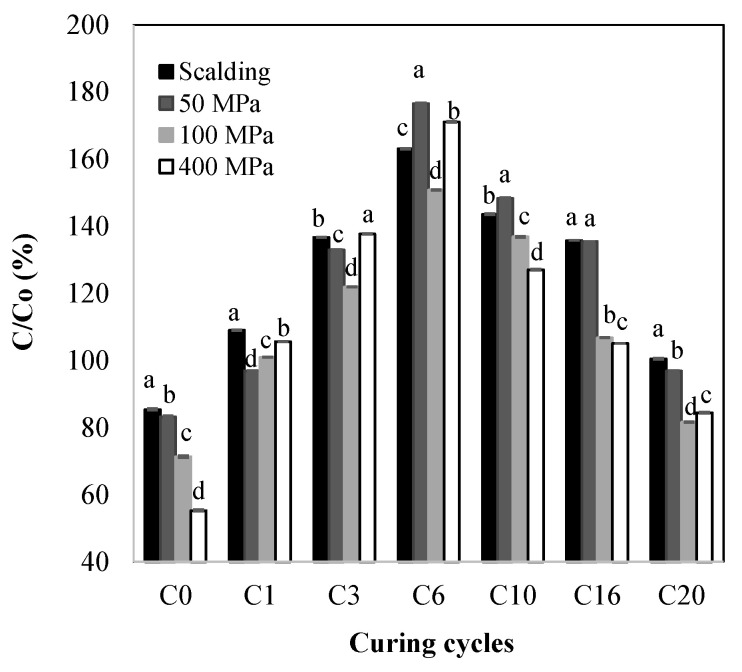
Relative total phenolic content (TPC) with respect to the untreated green beans (C0) at different curing cycles (C0–C20) for the high hydrostatic pressure-assisted cured and blanched beans. Different letters within each curing cycle indicate significant differences (*p* < 0.05).

Phenolic compounds are implicated in the color and flavor development of vanilla beans during curing. Throughout this process, the color of vanilla beans turns from green to dark brown. The change in the total phenolic content (TPC) evaluated immediately after (C0) the killing treatment at 50, 100, and 400 MPa for 5 min and during the subsequent curing cycles (C1–C20) was analyzed and compared with a traditional method for vanilla killing (blanching) (Figure 1). Immediately after vanilla killing, the blanched samples showed the highest phenolic content followed with the HHP treatment at 50 MPa. The blanched- and HHP-50-MPa-treated beans retained approximately 85 and 83% of the initial phenolic content of the untreated green beans (14.5 mg GAE/g), respectively, while the samples treated at 100 and 400 MPa retained approximately 71 and 55% of the original TPC, respectively. Once the beans were subjected to different cycles of sweating–drying (C1–C20), the TPC started to increase, reaching the maximum concentration at C6. At C6, the TPC increased by approximately 63, 77, 51, and 71% compared with the untreated beans for the blanched, 50, 100, and 400 MPa samples, respectively. After C6, the TPC started to decrease (Figure 1), reaching similar contents to those of the untreated green beans for the blanched- and 50-MPa-treated samples and retaining about 82% of the original content of the untreated green beans at 100 MPa and 84% at 400 MPa. Contrary to these results, Tapia–Ochoategui et al. [22] reported that the maximum concentration of vanillin was found after 10 curing cycles by applying a traditional method of curing that includes killing at 97 °C for 6–8 s.

Different effects on the TPC after blanching have been observed by other authors for various foods. Wen et al. [23] found increments up to 179% for four-angled bean and up to 32% for French bean (blanched with boiling water for 10 min). Oboh [24] reported for leafy green vegetables increments in the TPC from 0.1–0.3 g/100 g to 0.2–0.6 g/100 g after hot water blanching for 5 min. On the other hand, Wen et al. [23] found maximum reductions of 27 and 44% for blanched snow peas and snap peas, respectively. Blanching of carrots at 90 °C for 1 min resulted in a 20% loss of the original TPC [11]. The increase in phenolics due to thermal treatment has been attributed to the disruption of cell membranes and cell walls and the release of phenolics linked to other molecules which results in better extraction [23,25,26]. On the other hand, phenolic losses have been explained by autoxidation, breakdown, and by leaching in hot water during thermal treatment [11,27]. Just as with thermal treatments, the increment in the TPC in the pressurized samples could be attributed to cellular disruption and the release of phenolics from more complex cellular structures. It has been reported that pressure induces cell wall loosening by crosslinking or depolymerizing cell wall components, also affecting the cell membrane. Specifically, it has been observed that HHP affects membrane-bound or membrane-spanning proteins, in particular ion pumps or ion channels, which leads to increased membrane permeability [28,29,30]. HHP could improve the release of bioactive compounds from cellular compartments as follows: Once depolymerized from the cell wall, the membrane increases its permeability, which, consequently, includes changes in the intracellular concentrations of solutes [30]. During this exchange, phenolics are released by rupture of the vacuole, the main storage organelle of these compounds [28,29,30], which directly influences their extractability [31]. The increase in pressurized samples at pressures up to 100 MPa could also be related to the activation of the pathway for phenolic biosynthesis. It has been reported that at pressure levels lower than 100 MPa, the pressure could act as a stress factor activating the endogenous production of secondary metabolites such as phenolics [32]. Thus, the increment in the TPC at pressures < 100 MPa could be attributed to the metabolic stress produced by the HHP treatment. While the increment at pressures > 100 MPa could be attributed to metabolite release due to cell decompartmentalization [31]. These increments are favorable for vanilla curing due to the participation of phenolics in flavor development. However, to confirm the exact mechanism of the phenolic increment, microstructural studies, measurement of the phenylalanine ammonia lyase (PAL) activity, and gene expression, among other analyses, are needed.

The rate constant (*k*) for the increase in the TPC from C0 to C6 and decrease from C6 to C20 during the vanilla curing was determined using a first-order-type kinetic model (Table 1). The increase in TPC was characterized for *k* values of 0.102, 0.126, 0.113, and 0.164 days^−1^ for the blanching, 50, 100, and 400 MPa samples, respectively, with lineal correlation coefficients (R^2^) from 0.78 (400 MPa) to 0.98 (50 MPa). Despite the fact that the samples treated at 400 MPa showed the lowest TPC at C0, they presented the highest *k* value for phenolics increase, followed by 50 MPa, 100 MPa, and blanching. This suggests that HHP accelerates the rate of phenolic release or biosynthesis, as described above. A decrease in TPC was also fitted to a first-order-type kinetic model (R^2^ from 0.86 for blanching to 0.97 for 100 and 400 MPa) with *k* values of 0.0307, 0.0386, 0.0435, and 0.0476 days^−1^ for blanching, 50, 100, and 400 MPa, respectively. In this work, the highest decrease rate was observed for beans treated at 400 MPa followed by 100 MPa. This suggests a greater degradation rate at these pressure levels. This behavior was related to the lower TPC found at the end of curing (C20) for 400 and 100 MPa. On the other hand, the blanching treatments exhibited the lowest decrease rate of TPC followed by 50 MPa, which corresponds with the higher TPC found for these treatments at the end of curing. In addition, according to the kinetic constants, the rate of increase in TPC was approximately 3.3, 3.3, 2.6, and 3.5 times higher than the rate of decrease for blanching, 50, 100, and 400 MPa, respectively. Other authors, working with carrots and mango slices, have also fitted the TPC degradation to the first-order kinetic model [11,18].

### 2.2. Polyphenol Oxidase (PPO) and Peroxidase (POD) Activity

**Figure 2:**
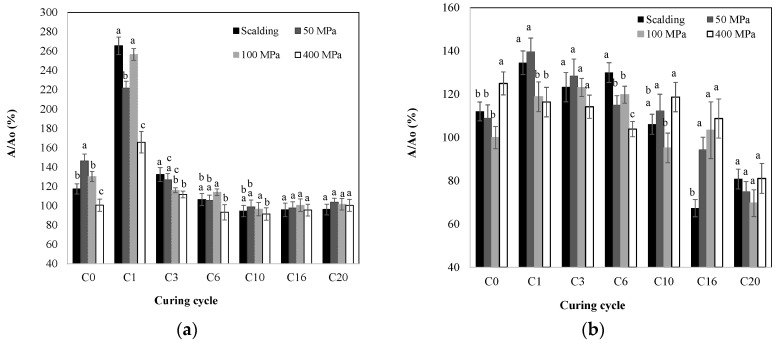
Relative (**a**) polyphenol oxidase (PPO) and (**b**) peroxidase (POD) activity with respect to the untreated green beans (*A_0_*) at different curing cycles (C0–C20) for the high hydrostatic pressure-assisted cured and blanched beans. Different letters within each curing cycle indicate significant differences (*p* < 0.05).

The change in the PPO and POD activity of the HHP and blanched vanilla beans at different curing cycles is presented in Figure 2. Figure 2a shows that immediately after killing, both blanching and HHP at 50 and 100 MPa increased PPO activity in about 17, 46 and 30%, respectively, when compared with the untreated green beans (166 μmol/min 100 mg), while for 400 MPa, no change in the PPO activity was observed. For both the blanched- and HHP-treated beans, the maximum PPO activity was detected in the first curing cycle of sweating–drying (C1), showing increases of approximately 165, 122, 156, and 66% for the blanching, 50, 100, and 400 MPa samples, respectively. No significant difference was found between the blanching and 100 MPa samples (*p* > 0.05). A comparison of the PPO activity at C0 with C1 showed that the rate of change in the PPO from C0 to C1 was higher for blanching followed by 100, 50, and 400 MPa.

Diverse mechanisms have been suggested for the increment in PPO activity by HHP. These include conformational changes and modifications in the reaction mechanisms, as well as in the substrate or solvent properties [33]. It has been reported that pressures lower than 100 MPa can activate some monomeric enzymes via conformational changes [34]. Waliszewski et al. [35] reported that the native PPO in vanilla beans is a monomeric enzyme; thus, this mechanism could explain the activation of PPO at C0 at 50 and 100 MPa. An increment in PPO activity has also been associated with cell decompartmentalization [34]. In this work, the increase in the PPO activity for the HHP at C1 and even for blanching at C0 and C1 could be attributed to the release of the enzyme from the bean tissue, which, once released, can interact with its substrate producing an increment in enzymatic activity. It has been reported that the promotion of the enzyme–substate interaction by PPH could result in either an increment or decrement of the catalytic activity depending on the activation volume that characterizes the specific system [34].

Figure 2a also shows that after the maximum activity, the PPO activity started to decrease, reaching a constant average activity from C10 to C20 of about 98, 101, 103, and 95% of the initial activity for blanching, 50, 100, and 400 MPa, respectively. No significant difference in the PPO activity at the end of curing was found among the treatments (*p* > 0.05). The reduction in the PPO activity could be related to the decrease in the water content and water activity with the progress of the curing cycles. In other works, it has been reported that after blanching at 70 °C/5 min, the beans retained less than 50% of the initial PPO activity of the untreated green beans, and after 36 sunny days they showed more than 80% of the initial PPO activity [1]. Discrepancies with this work could be attributed to the differences in the curing process and vanilla variety. The decrease in the PPO activity was characterized by *k* values of 0.106, 0.083, 0.090, and 0.064 days^−1^ for blanching, 50, 100, and 400 MPa, respectively; nevertheless, the statistical analysis indicated no significant difference (*p* > 0.05) among the *k* values for blanching, 50, and 100 MPa, while the treatment at 400 MPa had a *k* value approximately 1.7 times lower than the blanched beans (Table 2), meaning that at this pressure level, the rate of PPO activity loss was lower. This was related to the higher content of phenolics at the end of curing for blanching than for 400 MPa. An inverse relationship between the *k* for the TPC decrease and the *k* for reduction in the PPO activity for blanching and 400 MPa was observed. For blanching and 400 MPa, the highest the *k* for the TPC decrease, the lowest the *k* for the PPO activity decrease (Table 1 and Table 2). This is an expected behavior considering that the degradation of phenolics is higher when the rate for the loss of the PPO activity is lower. Thus, this lower loss of PPO activity at 400 MPa could explain the lower TPC found at C20 for samples treated at 400 MPa. In addition, the higher rate for PPO activity loss for the blanched vanilla is in accordance with the higher TPC for this sample at C20.

Analogously, the PPO inactivation in pear with thermal (45–75 °C) and HHP treatments (600 MPa/40–80 °C) [20] and in *Litopenaeus vannamei* treated with heat (60–100 °C) and HHP (400–600 MPa) [15] was described using a first-order model. The PPO inactivation of apple during high-humidity air impingement blanching followed a zero-order kinetic model at 90 and 100 °C and a first-order fraction model at 110 and 120 °C [12]. Zawawi et al. [19] reported a comprehensive review of the application of different kinetic models (including the first-order kinetic model) for the PPO inactivation using thermal, HHP, and ultrasound technologies in various fruits.

Regarding POD (Figure 2b), immediately after processing (C0), the activity for the blanched, 50, and 100 MPa samples was similar (*p* > 0.05), showing an average increase of about 7%, while for 400 MPa it increased approximately 25% compared with the untreated green beans (52.3 µmol/min 100 mg). This suggests an instantaneous release of POD from cellular structures after heating and pressurization that causes rupture of the cellular compartments where POD is located, leading to the enzyme running into the phenolic compounds released by the rupture of the vacuole (the main storage organelle of these compounds); thus, the interaction of the enzyme with its substrate increased the enzyme activity [36,37]. Unlike PPO, POD in vanilla is not a monomeric enzyme but rather a tetramer [38], so cell compartmentalization is the most probable mechanism for the increment in POD activity. As well as PPO, the maximum activity of POD was observed at C1, showing increments of 35, 40, 19, and 16 for blanching, 50, 100, and 400 MPa, respectively. At C1, no significant difference was found between blanching and 50 MPa or between 100 and 400 MPa (*p* > 0.05). Unlike PPO, POD activity did not reach a constant activity, rather it tended to decrease with the progress of the curing time. At the end of the curing (C20), a minimum average value of 77% of the initial PPO activity was reached, with no significant difference among treatments (*p* > 0.05). The preservation of high POD activity for all curing cycles suggests that the oxidation of phenolics occurs during the entire curing process of vanilla beans; thus, darker brown vanilla beans will be obtained at the end of curing. Cai et al. [1] observed that after the blanching of vanilla beans at 70 °C/5 min, they retained 97% of the initial POD activity of the untreated green beans, and after 36 sunny days they showed 19% of the initial POD activity. Again, discrepancies with this work could be attributed to the differences in the curing process and vanilla variety.

The rate constants for the decrement in the POD activity were calculated during the curing cycles from C1 to C20 using the first-order-type kinetic model. R^2^ values from 0.72 to 0.93 were obtained for blanching, 50, and 100 MPa, but the data for 400 MPa were not adequately described with the first-order model (R^2^ = 0.49) or any other model (data not shown). The highest *k* values for the decrease in POD activity were observed for the blanched- and 50-MPa-treated samples, with no significant difference (*p* > 0.05), meaning that for these treatments the loss of POD activity is faster and lower oxidation of phenolics occurs, which is related to the higher percentage of TPC found for these samples at the end of curing (Figure 1). The lowest decrease rate was found for 400 MPa, which was also related to the low TPC found for this treatment at C20. An inverse relationship between the rate of phenolic decrease and the loss of POD activity was found (R^2^ = 0.99). Thus, the higher the *k* value for the TPC decreases, the lower the *k* for the PPO activity loss, meaning that the enzyme still has high activity and, therefore, the rate of phenolic decrease is high. The rate of phenolic decrease could be related to the rate of flavor development. Nevertheless, complementary studies are needed to confirm the effect on the rate of formation and profile of vanilla flavor. Likewise, the inactivation of POD in blanched pumpkins has been reported to follow a first-order model [14]. The POD inactivation of seedless guava (*Psidium guajava*) cubes during the thermal treatment from 80 to 95 °C also followed a first-order kinetic model [39]. The inactivation of POD in sapodilla jam [16] and in strawberry puree [17] during the combined high-pressure–thermal processing was also well described by a first-order kinetic model.

### 2.3. Color Parameters

**Figure 3:**
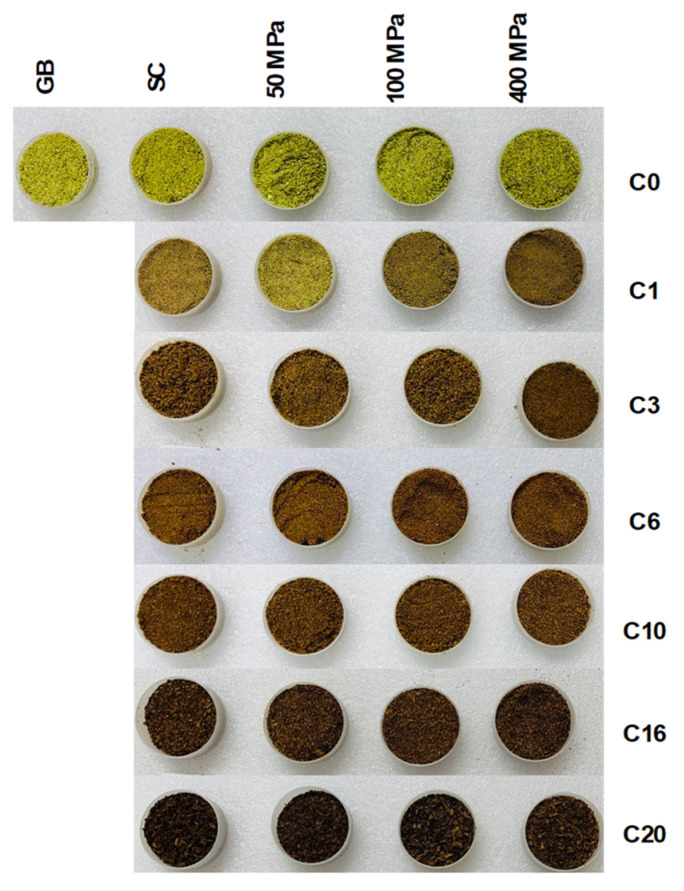
Visual color changes at different curing cycles (C0–C20) during the high hydrostatic pressure-assisted curing of vanilla beans compared with the blanched beans. GB = untreated green bean.

The visual color change during different curing cycles is presented in Figure 3. 

**Figure 4:**
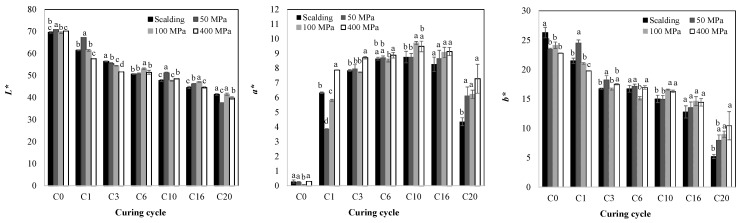
Changes in color parameters L*, a*, and b* at different curing cycles (C0–C20) during the high hydrostatic pressure-assisted curing of vanilla beans compared with the blanched beans. Different letters within each curing cycle indicate significant differences (*p* < 0.05).

As expected, it is observed in Figure 3 that for all treatment conditions, the color turns from green to dark brown. To analyze the color change in vanilla beans, the color parameters L*, a*, and b* were determined (Figure 4). 

Color values showed that before killing, green beans presented L*, a*, and b* values of 71.33, −0.37, and 23.99, respectively. These color parameters correspond to green (-a*) and yellowness colorations (+b*) with high luminosity. Immediately after the HHP and blanching (C0) treatments, all samples showed L* values similar to those of the green beans, while from C1 to C20, there was a decreasing trend, reaching a minimum value of 37.6 for 50 MPa, whereas 41.4, 41.5, and 39.8 were obtained for blanching, 100 MPa, and 400 MPa at C20. No significant difference was found between blanching and 100 MPa (*p* > 0.05). No correlation between L* and PPO, POD, or TPC degradation was found, suggesting that the change in color is also associated with nonenzymatic browning. Regarding the b* value, it increased slightly for the blanched beans at C0 (Figure 4), and like L*, it tended to decrease from C1 to C20, suggesting the destruction of yellow pigments. This reduction is attributed to phenolic browning reactions which produce polymeric dark brown pigments during vanilla curing, causing the transition from green to dark brown colors [40]. Immediately after curing, the a* parameter for both blanching and HHP changed from negative (greenness) to positive (redness) with values close to zero. At C1, a* was about 22, 16, 159, and 17 times higher than at C0. In addition, a* tended to increase with the progress of the curing time, reaching a maximum value of about 9.6 for 100 and 400 MPa and about 8.8 for the blanched and 50 MPa samples after 10 cycles of curing. At C16, a* started to decrease reaching a value of 4.4 for the blanched vanilla and an average value of 6.5 for the HHP-treated samples at C20. The decrement in a* at C20 could be related to the degradation of various pigments in the beans.

The transition from the negative a* value in the green beans to the positive value in the cured beans and its subsequent increase are attributed to the oxidation of chlorophyll during vanilla curing [40]. Before curing, chlorophyll (type *a* and *b*) is the main pigment in vanilla beans. During killing, the cell walls of the beans soften, and the chlorophyll starts to break down, contributing to the change in color. The decrease in chlorophyll *a* and chlorophyll *b* content is associated with their degradation into chlorophyll derivatives (pheophytin *a*, pyrophyllium chlorophyll *a,* and pheophorbide *a*), which are involved in browning, leading to a color change from green to brown [41,42]. In such a manner, once chlorophyll breaks down in vanilla beans, nonenzymatic browning will occur, and once the phenolic compounds are released, they also contribute to chlorophyll degradation (promoted by weak acids). Thus, color change in vanilla beans is attributed to both enzymatic and nonenzymatic browning [1]. During enzymatic browning, phenolic compounds are the substrate of the oxidative enzymes PPO and POD. In this work, both enzymes were highly active during all the curing cycles, especially at C1. It has been suggested that they act synergistically: while PPO in the presence of molecular oxygen (O_2_) generates hydrogen peroxide (H_2_O_2_) from the oxidation of vanillin and other phenolics, resulting in dehydrodivanillin, POD uses it as a substrate to produce dark brown pigments during enzymatic browning [43,44].

## 3. Materials and Methods

### 3.1. Vanilla Beans

Approximately 8 kg of mature green beans (*Vanilla planifolia*), harvested in February 2020 from Papantla, Veracruz, Mexico, was used in this study (lat. 20°09′–20°41′ N, long. 97°06′–97°32′ W, alt. 180 m).

### 3.2. High Hydrostatic Pressure (HHP)-Assisted Curing

Mature green vanilla beans were washed with tap water and divided into portions of 50 beans for each treatment. As a control sample, green beans were blanched by immersion in boiling tap water for 8 s to simulate the killing conditions used in situ in Papantla, Veracruz, Mexico. For the HHP treatment, the samples were placed in 11 × 8 cm polyethylene bags, vacuum-sealed, and treated in an HHP unit (H135, Hyperbaric, Burgos, Spain) using water at 7 °C as a pressure transmitting medium. Vanilla beans were treated at 50, 100, and 400 MPa for 5 min. After pressure treatment, vanilla beans were cured under 20 cycles in an incubator (KK400 TOP+/FIT P, POL-EKO, Vladislava, Poland) at controlled temperature, relative humidity (RH), and light. Each cycle, composed of sweating (45 °C/>90% RH) and drying stages (50 °C/60% RH/light) to simulate the curing conditions performed in situ in Veracruz, had a duration of 1 day. All through the vanilla curing, the beans were sampled as follows: cycle 0 (C0), cycle 1 (C1), cycle 3 (C3), cycle 6 (C6), cycle 10 (C10), cycle 16 (C16), and cycle 20 (C20). After being collected, the samples were frozen in liquid nitrogen and stored at −80 °C until analyzed [5].

### 3.3. Freeze Drying

Untreated green and cured vanilla beans were freeze-dried before analysis following the methodology described by Buitimea-Cantua et al. [5]. Frozen beans were ground using a food processor (Xpert Series, Oster, FL, USA) for 1 min at the highest speed level. The samples were subsequently stored at −80 °C for 3 h and, finally, freeze-dried at −55 °C, 0.04 mBar for 96 h (FreeZone Triad, Labconco, KS, USA). The freeze-dried vanilla beans were stored at −80 °C until analysis of the total phenolic content (TPC), polyphenol oxidase (PPO), and peroxidase (POD) activities and thes color.

### 3.4. Total Phenolic Content (TPC)

The TPC was determined following the methodology of Waterhouse with modifications [45]. Briefly, 100 mg of freeze-dried sample was mixed with 10 mL absolute ethanol. The mixture was kept under maceration for 24 h at room temperature. After, it was centrifuged (1764× g/10 min/25 °C), and the supernatant was filtered using a 0.45 µm PTFE membrane. Total phenolic extracts (50 μL) were mixed with 650 μL water, 50 μL Folin–Ciocalteu reagent, and 250 μL sodium carbonate (0.5 M). The mixture was incubated at 37 °C for 2 h. For the spectrophotometric analysis, 200 μL of the sample was transferred to a Costar 96-well flat, clear bottom microplate, and the absorbance was measured at 765 nm in a microplate reader (Synergy HT, BioTek Instruments, Bad Friedrichshall, Germany). A six-point calibration curve (R^2^ = 0.997) from 50 to 300 ppm of gallic acid (GA) was used to obtain the phenolic concentrations. The TPC is expressed as the relative content in percentage with respect to the untreated green beans on a dry basis (C/C_0_).

### 3.5. Polyphenol Oxidase (PPO) Activity

The PPO activity was determined in freeze-dried vanilla beans following the methodology of Vásquez-Caicedo et al. [46]. The enzyme extract was obtained by mixing 200 mg of the sample with 2.5 mL McIlvaine buffer solution (pH 6.7) consisting of 30% of 0.1 M citric acid and 70% 0.2 M sodium phosphate dibasic. The mix was homogenized in a vortex at 1000 rpm for 1 h at 4 °C and centrifuged (Mikro 220R, Hettich Instruments, Beverly, CA, USA) at 176× *g*/5 min/25 °C. The supernatant was centrifuged again (1313× g/10 min/4 °C) to obtain the enzyme extract. The reaction was started by mixing 210 μL of 0.5 mM sodium dodecyl sulfate (SDS), 30 μL enzyme extract, 30 μL of 0.5 M *L*-proline, and 30 μL of 25 mM 4-methyl-catechol solution. The absorbance was read at 525 nm at 25 °C for 1 h in one-minute intervals using a plate reader. The enzymatic activity was calculated using the Beer–Lambert law a = *ε*bc, with an extinction coefficient (*ε*) of 1550 L mol^−1^ cm^−1^, b = 1 cm, and the a = slope obtained from the linear portion of the curve resulting from plotting abs versus time. Enzymatic activity is expressed as the relative activity in percentage with respect to the untreated green beans (A/A_0_) on a dry basis.

### 3.6. Peroxidase (POD) Activity

The POD activity was determined in freeze-dried vanilla beans following the methodology of Dignum et al. [47]. Approximately 100 mg of freeze-dried vanilla sample was mixed with 10 mL of buffer extraction (sodium phosphate buffer, 0.1 M, pH 7). The mix was homogenized in a vortex for 5 min at 1500 rpm at room temperature and centrifuged (Mikro 220R, Hettich Instruments, Beverly, CA, USA) at 3000 rpm for 5 min at 25 °C. The supernatant was transferred to an Eppendorf tube and centrifuged at 12,500 rpm for 10 min at 4 °C to obtain the enzyme extract. The reaction was started by mixing 25 μL of 40 mM guaiacol, 50 μL enzyme extract, and 75 μL of 1.5 mM hydrogen peroxide, prepared in McIlvaine buffer pH 3.8. The absorbance was read at 470 nm at 25 °C for 3 min in 10 s intervals using a microplate reader. The enzymatic activity was calculated as described for the PPO using an extinction coefficient of 26,600 M^−1^ cm^−1^. Enzymatic activity is expressed as the relative activity in percentage with respect to the untreated green beans (A/A_0_) on a dry basis.

### 3.7. Color Parameter Measurement

Color in the freeze-dried vanilla beans was assessed using a handheld spectrophotometer (CM-600D, Konica Minolta, Tokyo, Japan). The equipment was calibrated with the standards provided by the supplier. The colorimetric parameters L*, which indicates lightness (varying from 0—black to 100—white); a*, which measures greenness (-a*)/redness (+a*); and b*, which indicates blueness (-b*)/yellowness (+b*), were measured five times in each sample [14].

### 3.8. Kinetic Analysis

The rate of the TPC, PPO, and POD change during the progress of the curing cycles (time in days) were analyzed using kinetic-type models. The phenolics and POD activities were fitted to the first-order kinetic-type model (Equation (1)), while the PPO was fitted to Equation (2). The rate constants (*k*) for the increment in the TPC from C0 to C6, as well as for the decrement for C6–C20, and for the decrement of the PPO and POD from C1 to C20 were calculated using the following equations [48]:

(1) $$\ln\left(\frac{C}{C_{0}}\right)=-kt$$

(2) $$ln\left(1+\frac{C}{C^{*}}\right)=-kt$$

where *C* represents the TPC concentration or enzyme activity (A) at each curing cycle, *C*_0_ represents the TPC or enzyme activity (*A*_0_) in the untreated green vanilla beans, and C* is the enzymatic activity at equilibrium.

### 3.9. Statistical Analysis

All analysis were performed in triplicate using independent samples, and each replicate was measured three times (*n* = 9). One-way ANOVA followed by the Tukey test (α = 0.05) was used to determine statistically significant differences between the HHP treatments and blanched beans. Statistical analysis was performed using the MINITAB 14.1 software version for windows^®^.

## Figures and Tables

**Table 1:** Rate constant (*k*, days^−1^) for the increase and decrease in total phenolic content (TPC) during the high hydrostatic pressure-assisted curing of vanilla beans and blanched beans.

		Blanched	50 MPa	100 MPa	400 MPa
TPC increment (C0–C6)	*k*	1.02 × 10^−1^ ± 1.79 × 10^−4 d^	1.26 × 10^−1^ ± 2.06 × 10^−4 b^	1.13 × 10^−1^ ± 2.43 × 10^−4 c^	1.64 × 10^−1^ ± 4.20 × 10^−4 a^
	R^2^	0.92	0.98	0.88	0.78
TPC decrement (C6–C20)	*k*	3.07 × 10^−2^ ± 6.93 × 10^−5 d^	3.86 × 10^−2^ ± 7.87 × 10^−5 c^	4.35 × 10^−2^ ± 9.72 × 10^−5 b^	4.76 × 10^−2^ ± 1.01 × 10^−4 a^
	R^2^	0.86	0.91	0.97	0.97

Different letters within the same row indicate significant differences (*p* < 0.05).

**Table 2:** Rate constant (*k*, days^−1^) for the decrement in polyphenol oxidase (PPO) and peroxidase (POD) activity during the high hydrostatic pressure-assisted curing of vanilla beans and blanched beans.

		Blanched	50 MPa	100 MPa	400 MPa
PPO (C1–C20)	*k*	0.106 ± 0.012 ^a^	0.083 ± 0.011 ^a,b^	0.090 ± 0.007 ^a,b^	0.064 ± 0.019 ^b^
	R^2^	0.82	0.83	0.66	0.88
POD (C1–C20)	*k*	0.034 ± 0.001 ^a^	0.029 ± 0.001 ^a,b^	0.025 ± 0.003 ^b^	0.014 ± 0.002 ^c^
	R^2^	0.81	0.93	0.72	0.49

Different letters in the same row indicate significant differences (*p* < 0.05).

## Data Availability

Data is contained within the article.
